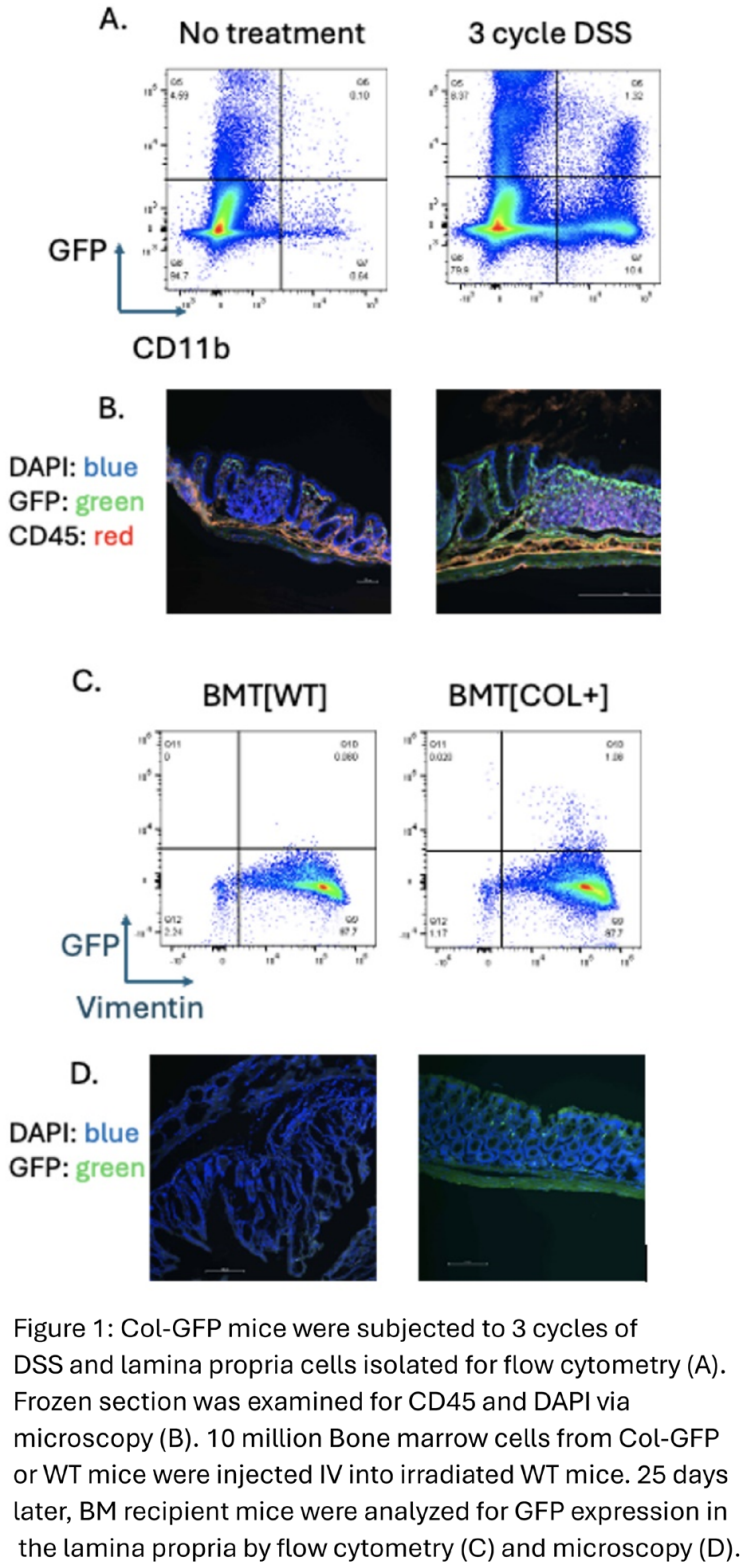# Poster Session I - A27 COLLAGEN-PRODUCING, GFP-TAGGED BONE-MARROW DERIVED CELLS MIGRATE TO COLONIC LAMINA PROPRIA AND MAY CONTRIBUTE TO INTESTINAL FIBROSIS IN A MURINE MODEL OF COLITIS

**DOI:** 10.1093/jcag/gwaf042.027

**Published:** 2026-02-13

**Authors:** R R Peng, E Pearce, A Ueno, Y Li, H B Jijon, P L Beck

**Affiliations:** University of Calgary Cumming School of Medicine, Calgary, AB, Canada; University of Calgary Cumming School of Medicine, Calgary, AB, Canada; University of Calgary Cumming School of Medicine, Calgary, AB, Canada; University of Calgary Cumming School of Medicine, Calgary, AB, Canada; University of Calgary Cumming School of Medicine, Calgary, AB, Canada; University of Calgary Cumming School of Medicine, Calgary, AB, Canada

## Abstract

**Background:**

Intestinal fibrosis is a common and severe complication of Crohn’s disease (CD). A central feature is the deposition of collagen resulting in stiffening and narrowing of the bowel.

Fibrocytes are a bone marrow (BM)-derived, CD45+, CD11b+. vimentin- and collagen-expressing cells contributing to fibrosis in multiple organs such as the lung and liver. We previously reported an increase of fibrocytes in blood and fibrostenotic tissue from CD patients. However, the origin of these cells, presumed fibrocytes, remains unknown.

**Aims:**

In this study, we use GFP-collagen I (Col-GFP) reporter mice to assess whether BM-derived, collagen-expressing cells (putative fibrocytes) contribute to intestinal fibrosis in an animal model of colitis.

**Methods:**

Col-GFP mice were subjected to 3 cycles of DSS to trigger intestinal fibrosis in the context of colitis. Colitis and fibrosis in DSS-treated mice were assessed after 3 cycles of DSS and recovery. Ten million bone marrow cells from Col-GFP mice and WT (GFP-) control mice were transferred into WT, irradiated, congenic mice via intravenous injection. The recipient mice were analyzed for the presence of GFP-expressing cells 25 days post-bone marrow transplantation (BMT). Colonic lamina propria were analyzed by flow cytometry and immunofluorescence.

**Results:**

Col-GFP mice demonstrate a marked increase in intestinal GFP expression following 3 cycles of DSS (Figure 1 A,B). Following BMT, Col-GFP recipient mice demonstrate the presence of GFP+ cells in colon lamina propria, relative to WT recipient mice (7.00% +/- 2.21, n = 3 vs. 0.06% +/- 0.03, n = 6, *p = 0.02*). This is supported by immunofluorescence imaging (Figure 1 C,D).

**Conclusions:**

Cyclical exposure to DSS elicits intestinal fibrosis in Col-GFP mice, demonstrating this is a suitable model to explore the cellular source(s) leading to collagen deposition during intestinal fibrosis. Furthermore, bone marrow-derived, collagen-expressing GFP+ cells migrate to the colon under homeostatic conditions. This suggests that, in addition to local mesenchymal cells, bone marrow-derived cells may contribute to intestinal collagen deposition in health and disease. Future experiments will assess the contribution of BM-derived cells to collagen deposition following cyclical DSS treatment of BM chimeras.

**Funding Agencies:**

CIHR